# Unveiling Semiconductor Nanostructured Based Holmium-Doped ZnO: Structural, Luminescent and Room Temperature Ferromagnetic Properties

**DOI:** 10.3390/nano11102611

**Published:** 2021-10-04

**Authors:** Guy L. Kabongo, Gugu H. Mhlongo, Mokhotjwa S. Dhlamini

**Affiliations:** 1 Department of Physics, University of South Africa, P.O. Box 392, Pretoria 0003, South Africa; dhlamms@unisa.ac.za; 2 CSIR-National Centre for Nano-Structured Materials, P.O. Box 395, Pretoria 0001, South Africa; gmhlongo@csir.co.za

**Keywords:** ZnO, holmium, nanostructures, ferromagnetism, XPS

## Abstract

This research work describes the synthesis of ZnO nanostructures doped with Ho^3+^ ions using a conventional sol–gel synthesis method. The nanostructured produced exhibited a wurtzite hexagonal structure in both ZnO and ZnO:Ho^3+^ (0.25, 0.5, 0.75 mol%) samples. The change in morphology with addition of Ho^3+^ dopants was observed, which was assigned to Ostwald ripening effect occurring during the nanoparticles’ growth. The photoluminescence emission properties of the doped samples revealed that Ho^3+^ was emitting through its electronic transitions. Moreover, reduced surface defects were observed in the Holmium doped samples whose analysis was undertaken using an X-ray Photoelectron Spectroscopy (XPS) technique. Finally, enhanced room temperature ferromagnetism (RT-FM) for Ho^3+^-doped ZnO (0.5 mol%) samples with a peak-to-peak line width of 452 G was detected and found to be highly correlated to the UV–VIS transmittance results.

## 1. Introduction

**Scheme 1:**
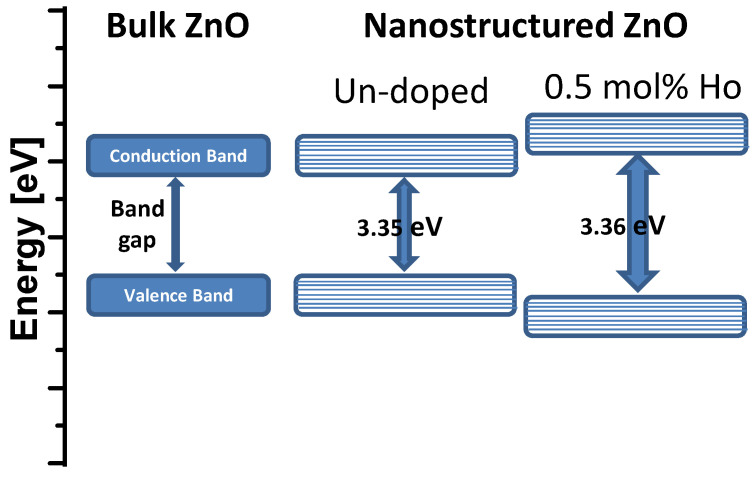
ZnO Band-gap engineering.

The surge of interest observed on ZnO nanostructures research in the past three decades among the scientific community was due to the versatile optical, magnetic and surface properties that they display under room temperature conditions [1]. The ZnO wide direct tunable band-gap, large exciton binding energy at room temperature makes it to be a convenient material for device fabrication in a wide range of applications such as optoelectronic, photonics and dilute magnetic semiconductors (DMS) [2,3,4,5]. It has potentialities to be applied in other devices such as gas sensors [6], biolabels [7], solar cells [8] and piezoelectric nanogenerators [9]. Recently, several research groups have investigated the optical properties of ZnO nanostructures exhibiting different morphologies such as flower-like [10], nanorods [11], comb-like [12] and nanowire arrays [13]. The need to engineer ZnO defects has driven scientists to dope this semiconductor material with either transition metal ions or light emitting elements such as the promising rare earth ions which in most cases resulted in tailoring the band-gap of ZnO [14]. Despite its large magnetic moment, Ho^3+^ has attracted less interest within the scientific community. Recently, Popa et al. [15] investigated the impact of structural on optical properties of sol-gel derived holmium doped ZnO thin films using relatively higher doping concentrations (1, 3, 5 at%), and others have demonstrated elsewhere that holmium concentration variation is very effective in tuning ZnO properties [16]. Earlier, Khataee and co-workers [17] reported on holmium doped zinc oxide nanoparticles synthesized via sonochemistry for the evaluation of the Reactive Orange 29 degradation in catalysis. Moreover, Singh and colleagues have successfully measured the DC magnetization and resistivity in Ho doped ZnO nanoparticles produced via wet chemical synthesis [18]. Furthermore, in their investigation dedicated to unveiling the optical and dielectric properties of Ho-doped ZnO, a research group have successfully established the correlation of the abovementioned properties to ZnO related defects [19]. Interestingly, sol–gel remains to date one of the most efficient synthesis procedure to produce ultrasmallZnO nanoparticles [20,21]. In the current study, the observed change in morphology in Ho^3+^-doped ZnO nanocrystals is reported, and it was assigned to Ostwald ripening effect which occurred during the growth process of ZnO nanostructures. Moreover, a comparison of the defect state XPS core levels in un-doped and Ho^3+^-doped ZnO (0.5 mol%) samples is presented and discussed in detail. Furthermore, in spite of the low concentration of Ho^3+^ dopant, XPS Ho 4d core levels were detected. Finally, the current research work is a novel experimental study on room temperature ferromagnetism based on microwave absorption of Ho^3+^ ions doped in ZnO nanostructures owing to the large magnetic moment of Ho^3+^. The ferromagnetism enhancement observed which is related to the number of spins participating to the ferromagnetic resonance is found to be in accordance with the UV–VIS transmittance and band-gap results (Scheme 1).

## 2. Experimental Section

The samples used in the present study were all synthesized in the same conditions, except the molar concentration of Ho dopant which varied. The synthesis was conducted using zinc acetate (Zn(CH_3_COO)_2_·2H_2_O), sodium hydroxide (NaOH) and holmium nitrate pentahydrate (Ho(NO_3_)_3_·5H_2_O), which were all purchased from Sigma-Aldrich (Kempton Park, Gauteng, South Africa) and used as received. ZnO:Ho^3+^ nanocrystals were synthesized by a sol–gel method following the same procedure as reported in our previous works [22,23,24]. The solution of sodium hydroxide dissolved in ethanol was prepared separately, then cooled in ice water and added dropwise judiciously to the ethanol solution of Zn^2+^ ions. For preparation of Ho^3+^-doped ZnO samples with different concentrations of Ho^3+^ (0.25, 0.5 and 0.75 mol%), the ethanol solution of Holmium nitrate pentahydrate was added into the hydrolyzed Zn^2+^ solution prepared following the above route. The obtained clear solution was kept at room temperature for 24 h and then washed several times in a mixture of ethanol and heptane (1:2 molar ratio) to eliminate unreacted Na^+^ and CH_3_COO^−^ions. The resulting precipitates were then re-dispersed in ethanol and dried at 200 °C for 2 h in an electric oven (ambient atmosphere).

The structure of the obtained samples was characterized on an X’Pert PRO PANalytical diffractometer with CuKα at λ = 0.15405 nm. Transmission electron microscopy (TEM) images were taken by using a JEOL-Jem2100 microscope. Morphology and chemical composition of the samples (Cu-grid deeped in ethanolic solution of the sample) were analyzed using a JEOL JSM-7500F field-emission scanning electron microscope (FE-SEM, JEOL Ltd.,Tokyo, Japan) equipped with energy dispersive X-ray spectrometer (EDX) (an Oxford Instruments, High Wycombe, UK). Photoluminescence (PL) and Time Resolved PL properties for the un-doped and doped samples were analyzed using a JobinYvonFluorolog 3 spectrofluorometer equipped with a Xenon lamp and nanoLED for excitation at room temperature. The transmittance measurements were carried out using a Perkin-Elmer Lambda 1050 UV/Vis/NIR spectrophotometer. The X-ray photoelectron spectroscopy (XPS) core levels were carried out using a PHI 5000 Versaprobe-Scanning ESCA Microprobe (ULVAC-PHI, Inc. Kanagawa, Japan). Finally, the room temperature magnetization measurements were examined through the microwave absorption measurements collected using a JEOL X-band electron spin resonance (ESR; JEOL Ltd.,Tokyo, Japan) spectrometer (JES FA 200; JEOL Ltd.,Tokyo, Japan) operating at 9.4 GHz equipped with an Oxford ESR900 gas-flow cryostat and a temperature controller (Scientific instruments 9700; Oxford Instruments plc, Abingdon, UK). In order to evade saturation during measurements, the microwave power was maintained at 5 mW. The DC static field HDC was slowly swept between 0 and 8360 Gauss. The DC field was modulated with an AC field whose amplitude was kept constant at 100 kHz frequency. The microwave absorption output was measured as a derivative signal.

## 3. Results and Discussion

### 3.1. EDS, SEM, TEM, XRD Analysis

**Figure 1:**
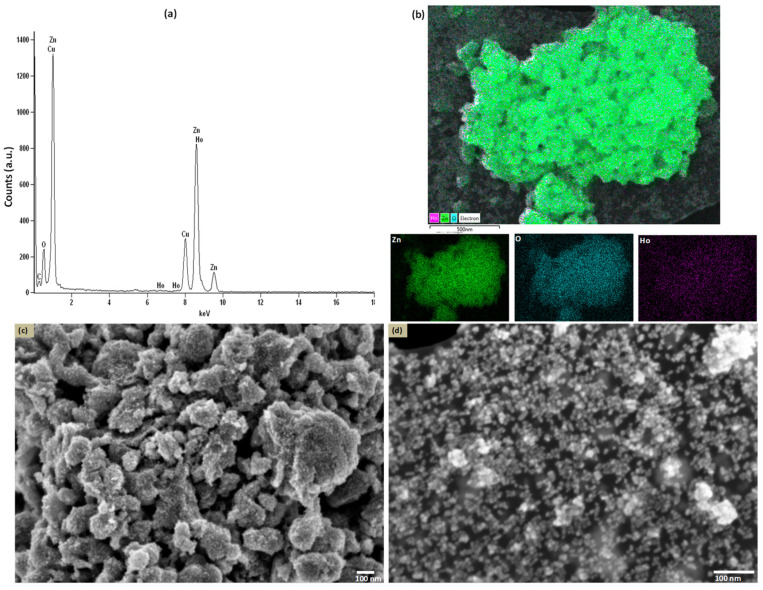
EDS (**a**) spectrum and (**b**) elemental mapping for 0.5 mol% Ho^3+^-doped ZnO nanocrystals. SEM micrographs for (**c**) un-doped ZnO and (**d**) (0.5 mol%) Ho^3+^-doped ZnO nanocrystals.

Considering that during the course of experiment the photoluminescence optimization which revealed that optimum luminescence was obtained from 0.5 mol% Ho^3+^-doped ZnO, particular attention was devoted to elucidating the microstructural behavior of 0.5 mol% Ho^3+^-doped ZnO sample relative to the un-doped ZnO. The elemental composition of the sample in Figure 1a was surveyed by EDS to demonstrate qualitatively the presence of holmium dopant in the investigated area of the sample in addition to zinc and oxygen. However, the observed copper peak originates from SEM sample preparation (i.e., Cu-grid) [14]. It is worth mentioning that the expected chemical species were all found to be well distributed throughout the analyzed area (see Figure S1) of the as-synthesized sample as shown in Figure 1b. Figure 1c,d shows classical SEM micrographs for the un-doped and 0.5 mol% Ho^3+^-doped samples, respectively. The images show that spherical-like ZnO particles in the case of un-doped sample and rod-like particles in the case of 0.5 mol% Ho^3+^-doped samples were formed during the wet chemical synthesis route. Interestingly, the observed mutation of particle morphology from un-doped to 0.5 mol% Ho^3+^-doped ZnO samples is attributed to the Ostwald ripening effect [25,26].

**Figure 2:**
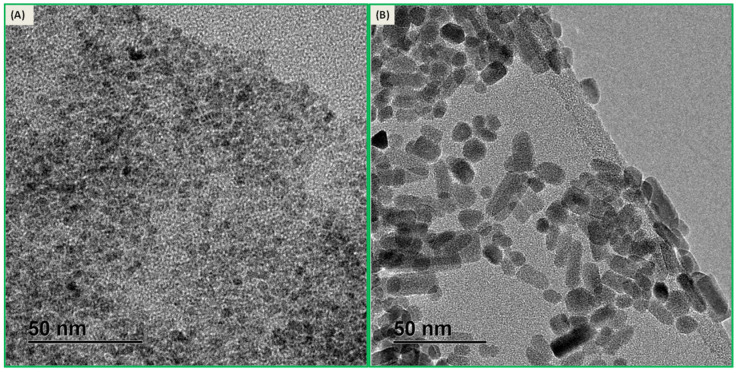
TEM images for (**A**) un-doped and (**B**) (0.5 mol%) Ho^3+^-doped ZnO nanocrystals.

TEM measurements were undertaken carefully on both un-doped and 0.5 mol% Ho^3+^-doped ZnO samples (see Figure 2). The measurements revealed that the nanostructures were evenly distributed and were highly crystalline. However, a peculiar phenomenon based on the mutation of morphology was observed and was attributed to doping with holmium ions, more precisely due to the growth of nanoparticles in solution through a diffusion limited Ostwald ripening process known to be the most predominantly growth mechanism so far [27]. However, further investigations are required in order to effectively elucidate on the observed mechanism of morphology mutation. It is however suggested that studies on colloidal nanocrystals growth under TEM using the so-called liquid cell electron microscopy [28,29] should be performed. Such study has been undertaken previously but not quite intensively, especially on the ZnO nanoparticles growth [30]. TEM analysis revealed that the particles diameter and width were ~10 nm for un-doped and 0.5 mol% Ho^3+^-doped ZnO, respectively, the former contained a mixture of spherical like and rod-like particles. The TEM result is found to be consistent with former SEM particles morphology characterization.

**Figure 3:**
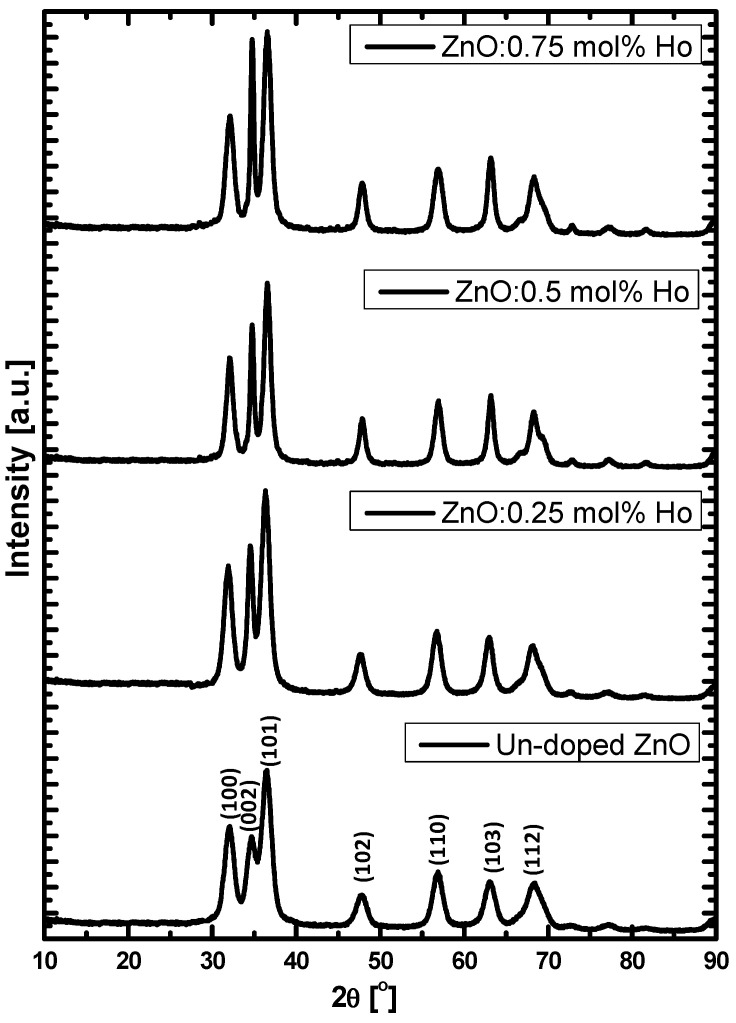
XRD patterns for undopedZnO and Ho^3+^-doped ZnO nanocrystals dried at 200 °C.

Figure 3 illustrates the X-ray diffraction (XRD) profiles of the ZnO and ZnO:Ho^3+^ nanocrystals. The analysis revealed that the as-synthesized nanocrystals ranging between 4–8 nm in diameter (See Table 1) were highly crystalline and exhibited the hexagonal wurtzite structure (space group *P*6_3_*mc*) indexed to JCPDS card # 36-1451. Furthermore, no second phase originating from Ho_2_O_3_ was observed in the Ho^3+^-doped ZnO samples [31], which confirms that the dopants successfully substituted the Zn^2+^ ions within the ZnO lattice structure. The Scherrer equation was employed to estimate the crystallite size [32];

(1) $$D=\frac{k\lambda }{\beta \cos Ө}$$

where *D*, *λ*, *β*, Ө and *k* are the crystallite size, the wavelength of the incident X-ray CuK radiation (0.15405 nm), the full width at half maximum (FWHM), the diffracting angle and a numerical constant (0.89), respectively. The results obtained are consistent with the TEM results, where the particles were in the nanometer range (see Figure S2).

### 3.2. UV–VISTransmittance and Photoluminescence for Undoped and Ho^3+^-Doped ZnO Nanostructures

**Figure 4:**
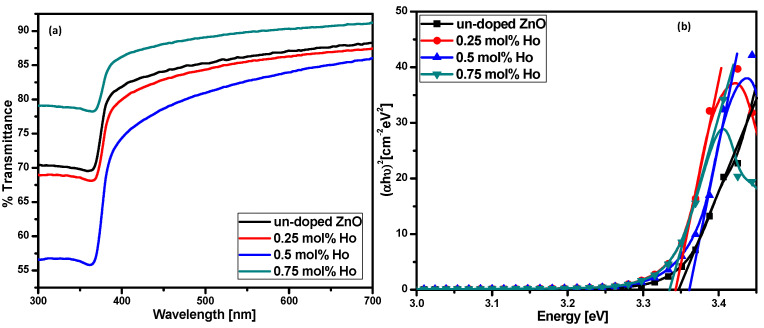
(**a**) UV–VIS transmission spectra for un-doped and Ho^3+^-doped ZnO nanostructures (**b**) optical band–gap extrapolation from Tauc plot.

The transmittance spectra in the range of 300–700 nm are depicted on Figure 4a. A sharp UV cut off band at approximately 360 nm was observed and assigned to the band-to-band transition of ZnO. The bandedge peak is found to be highly blue shifted as compared to the bulk ZnO (~386 nm) [33] owing to quantum confinement effect [34]. The un-doped ZnO sample exhibited a transmittance of the order of 40% far below the 0.5 mol% Ho^3+^ tramittance which is in the order of 60%. This implies that the incorporation of Ho^3+^ dopant state within the band-gap of the ZnO matrix has enhanced the transparency of the material. However, the transmittance was dropped after incorporation of 0.75 mol% Ho^3+^ defects owing to the segregation of the dopant. The optical band-gap (Figure 4b) was extrapolated using the Tauc’s formula [35]:

(2) $$\alpha \left(x\right)h\nu =A{\left(h\nu -E_{g}\right)}^{\frac{1}{2}}$$

where *A* is the constant, *hν* is the photon energy, *E_g_* the optical band-gap, and α(*x*) is the absorption coefficient of ZnO nanoparticle. The absorption coefficient can be calculated from the Beer–Lambert law:

(3) $$\alpha \left(x\right)=-\frac{1}{d}\ln\left(T\right)$$

where *T* is the normalized transmittance. The band-gaps were obtained from the Tauc plot depicted in Figure 4b. The extrapolation revealed optical band-gaps of~3.35, 3.34, 3.36 and 3.33 eV corresponding to un-doped ZnO, 0.25, 0.5 and 0.75 mol% Ho^3+^-doped ZnO, respectively. These obtained band-gaps values are in good agreement with previous reports [36].

**Figure 5:**
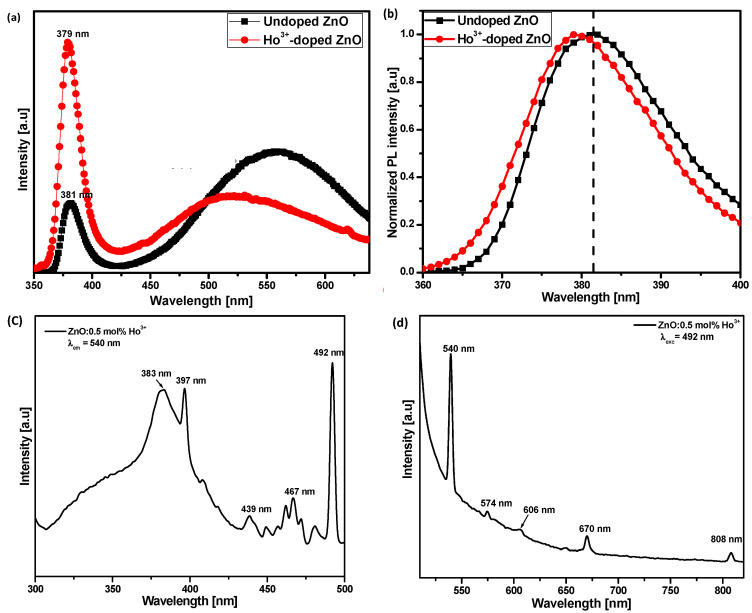
(**a**) Emission spectra for as prepared un-doped and 0.5 mol% Ho^3+^-doped ZnO samples (λ_exc_ = 325 nm), (**b**) shows the magnification of the normalized exciton emission peak. (**c**) Excitation and (**d**) emission spectra for 0.5 mol% Ho^3+^-doped ZnO nanocrystals.

Based on the previous characterization, the emission spectrum of the as-synthesized 0.5 mol% Ho^3+^-doped ZnOnano-phosphor sample was first examined by exciting with 325 nm electromagnetic radiations using a xenon lamp. It was found that Ho^3+^ (0.5 mol%) doped sample exhibited the optimum emission intensity. Figure 5a compares the PL spectra for both un-doped and 0.5 mol% Ho^3+^-doped ZnO samples using 325 nm (3.82 eV) UV light radiations for excitation. The most common reported ZnO defects emission trend was observed, and the exciton emission owing to the recombination of electron-hole pairs was also detected at 381 nm (3.25 eV) for the un-doped ZnO sample, while 379 nm (3.27 eV) was observed in the case of 0.5 mol% Ho^3+^-doped ZnO [37]. It is worth noting that after doping with Ho^3+^ ions, the free-exciton emission in the ZnO matrix undergoes two different kinds of modifications. The major alteration observed in the free-exciton emission was the slight blue shift (2 nm) observed to be derived from Ho^3+^doping in the ZnO matrix as compared to un-doped ZnO, and this shift implies a change in the band structure [38,39,40] (see Figure 5b). Elilarassi et al. [41] elaborated on such phenomenon, which was assigned to a decrease of the transition probability taking place within the band-gap of the doped ZnO matrix among the oxygen vacancy and the Zn vacancy after the substitution of Zn^2+^ ions by the dopant in the host lattice [39,40]. The second modification observed was the severe increase in intensity of the free-exciton emission observed with holmium doping, which was attributed to energy transfer from the Ho^3+^ ions to ZnO host and/or to the reduction in concentration of oxygen defects as reported in our previous study [14]. The observed enhancements of the exciton emission confirmed the ability of spontaneous lasing action in the ultraviolet spectral range, thus, positioning 0.5 mol% Ho^3+^-doped ZnO nanostructures as a candidate material for spintronic applications (see Figure 5a) [42,43].

Considering the current extensive controversies about the origin of the visible emission in ZnO, one can speculate on the defects that caused this emission [44]. A change in surface defect occurring on the surface of ZnO owing to Ho^3+^ doping could be the main factor causing the blue shift observed in the visible emission. Furthermore, the doping with holmium has occasioned band bending at the surface of ZnO resulting from the change of the Fermi energy level [45,46,47]. It is however important to undertake further investigation to elucidate this hypothesis.

Moreover, the ZnO:Ho^3+^ (0.5 mol%) emission and excitation spectra are depicted in Figure 5c,d. Five prominent excitation peaks at 383, 397, 439, 467 and 492 nm were observed from the spectrum. From these excitation wavelengths, we have selected the 492 nm wavelength to measure the emission spectrum of 0.5 mol% Ho^3+^-doped ZnO. A number of emission bands are detected spreading from the visible to the near infrared (NIR) regions at 540 (^5^S_2_,^5^F_4_→^5^I_8_), 606 (^5^F_5_→^5^I_8_), 670 (^5^F_5_→^4^I_8_), 757 (^5^S_2_,^5^F_4_→^5^I_7_) and 808 (^5^I_5_→^5^I_8_) nm (Figure 5d) [48,49,50,51,52]. The most prominent emission is observed in the green region centered at 540 nm with a less intense peak at about 574 nm; both peaks aredue to the hypersensitive (^5^S_2_,^5^F_4_→^5^I_8_)transition.

### 3.3. Surface State XPS Characterization

**Figure 6:**
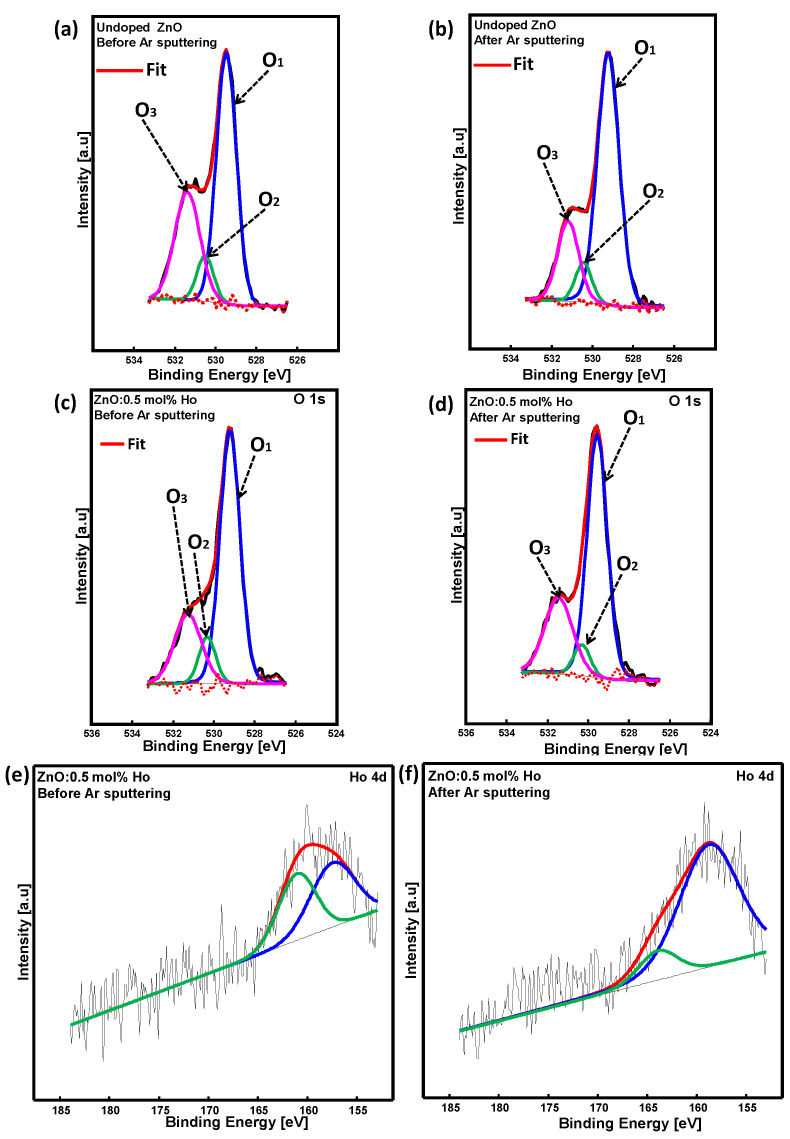
XPS O 1s core levels spectra for un-doped ZnO (**a**) before and (**b**) after Ar^+^ sputtering and 0.5 mol% Ho^3+^-doped ZnO (**c**) before and (**d**) after Ar^+^ sputtering. XPS Ho 4d core levels (**e**) before and (**f**) after Ar^+^ sputtering.

Figure 6 shows the XPS spectra for both un-doped and 0.5 mol% Ho-doped samples, which allow the elucidation of the oxidation state of different species present in the as-prepared nanostructures, namely, Zinc, Oxygen and Holmium. The survey scans (not presented here) for both samples were identical at the sole exclusion of Ho4d core level present in the Ho^3+^-doped ZnO samples. Besides Zn 2p, O 1s, and Ho 4d core levels, a C 1s core level arising from extrinsic surface impurities was also detected. The binding energy correction was applied using the C 1s (284.8 eV) core level. For both samples, the Zn 2p core level which appears as a doublet due to the interaction of the spin and orbital magnetic moments did not encounter any peak shift (not shown here). This observation implies that zinc is not sparingly sensitive to a change in the oxidation state [53]. The de-convoluted O 1s core level for (0.5 mol%) Ho^3+^-doped sample (see Figure 6) exhibited three oxygen components. The peaks appeared at 529.21 ± 0.05, 530.32 ± 0.05 and 531.27 ± 0.05 eV ascribed to O^2−^ ions on the hexagonal wurtzite structure of ZnO; O^2−^ ions in oxygen deficient regions within the ZnO matrix and chemisorbed species on the surface of ZnO, respectively, were observed [10]. As compared to the un-doped sample, the intensity of the O_2_ peak was found to decrease owing to a decrease in concentration of oxygen defects in the 0.5 mol% Ho^3+^-doped ZnO sample. This finding has a direct implication in the luminescence quenching of the green emission observed in the PL spectra, which was also demonstrated in our previous report [14] and similarly by Kumar et al. [54].However, the surface cleaning with Ar^+^ sputtering has an influence on the intensity of O_3_ peak, which is slightly decreased as compared to the peak before Ar^+^ cleaning. Furthermore, it is important to focus on the detection of Ho 4d core level peak at about 160.09 ± 0.05 eV binding energy (Figure 6e) [55]. The Ho 4d core level was de-convoluted and displayed two bands at about 161.04 ± 0.05 eV and 163.93 ± 0.05 eV before Ar^+^ sputtering. It is worth noting that the small shoulder appearing at about 175 eV could be assigned to multiplets splitting.

### 3.4. Room Temperature Ferromagnetic (RT FM) Properties

Being one of the most reliable spectroscopic techniques to probe defects at the surface and interface of materials, the ESR technique was used in the current study. ZnO is naturally diamagnetic even though there is still a controversial debate on the matter among scientists [56,57]. However, most scientific reports admitted the probability to observe two types of paramagnetic behaviour in ZnO. The low-field signal often assigned to unpaired electron trapped at an oxygen vacancy site (g = 2.0023) and the high-field signal owing to shallow donor centers (g = 1.96) [58]. The spectroscopic g-factor of the free electron can be calculated from the following equation:

(4) $$g=\frac{h\nu }{\beta B}$$

where *ν* is the microwave frequency, *h* is Planck’s constant, *β* is the Bohr magneton, and B is the magnetic field.

**Figure 7:**
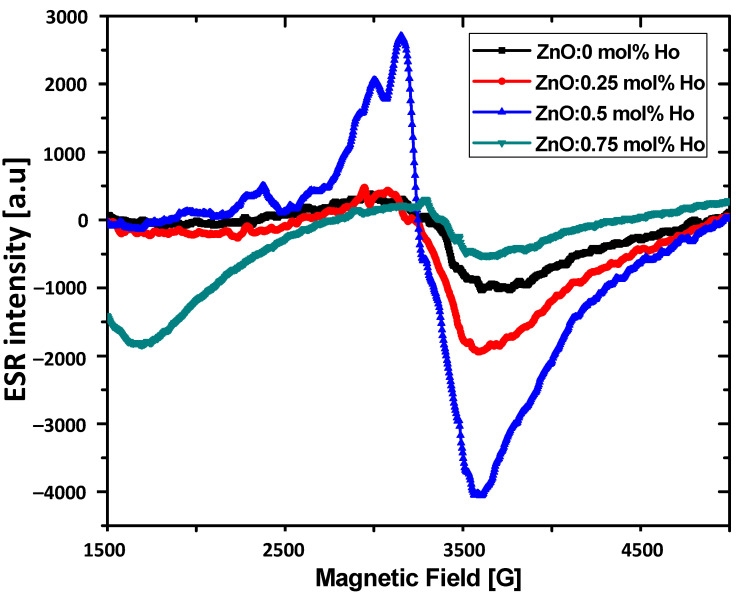
X-band (9.4 GHz) ESR spectra for un-doped and Ho^3+^-doped ZnO samples at room temperature.

The investigation of the magnetic behavior of Ho^3+^-doped ZnO nanostructures was successfully undertaken in this study. Due to the high sensitivity of the technique to defects, we conducted measurements on various concentrations in order to completely understand the effect of the doping concentration on the number of spins. It is a well-known fact that a number of non-magnetic materials have been observed to exhibit ferromagnetic properties at the nanoscale [10,59,60,61,62,63]. Scientists worldwide have found interest in investigating the RT-FM in un-doped and Ho^3+^-doped ZnO nanostructures [64,65]. It is worth mentioning that the *4f* rare earth elements exhibit exceptional magnetic properties as compared to the *4d* transition metal (TM) elements [66,67]. However, few studies reported the RT-FM of Ho^3+^ into ZnO[68,69,70,71]. At first sight, the ESR results obtained in the current investigation seem not to correlate well with the PL due to the fact that the Ho^3+^ doping into ZnO have induced a change in the nature of defects within the matrix. A similar trend was previously reported by Garcia et al. [72] who investigated the *d*^0^ ferromagnetism in ZnO capped with organic molecules. He hypothesized that the organic species present in ZnO samples could induce ferromagnetic-like behaviour (see Figure 7).

**Figure 8:**
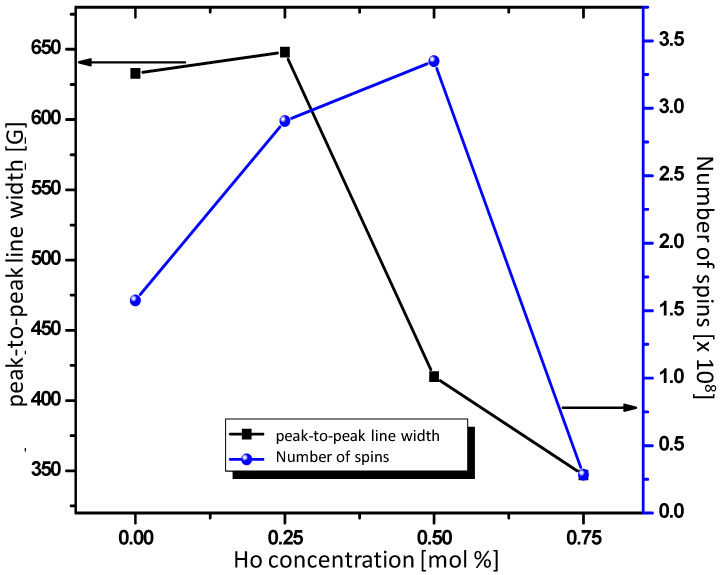
Peak-to-peak line width and number of spins as a function of Ho^3+^ concentration.

In spite of an abundant literature on the study of the RT-FM mechanism in ZnO nanostructures, the debate on its origin is still open and promising. Xiaoyong Xu et al. [73] have reported on the size dependence RT-FM in ZnO quantum dots; in their study they established a correlation between the RT-PL and the RT-FM in un-doped ZnO nanoparticles, denoting that the *d*^0^ ferromagnetism is largely related to the concentration of native defects such as oxygen vacancy in the samples. However, contrary to previous studies on un-doped ZnO, the doping with Ho^3+^ could be responsible for the magnetic intensity enhancement observed in this study which is not in accordance with the correlated PL/FM results reported elsewhere [73,74]. This observation is probably due to the important magnetic moment of the Ho^3+^ ions dopants, which effectively dominated on the RT-FM as compared to the concentration of oxygen defects. From the current observation and based on previous studies, we can speculate on the strong RT-FM observed in the Ho^3+^-doped samples which could be ascribed to the *s-f* coupling between the ZnO host and the Ho^3+^ dopant due to its large magnetic moment and the incorporation of more ferromagnetic defects within the ZnO matrix [63,64,66,75]. The collected ESR spectra revealed the occurrence of strong microwave absorption at about 3248 Gauss which may be assigned to unpaired electron trapped at an oxygen vacancy site. The obtained g-factor values corresponding to the ferromagnetic resonance of the un-doped and 0.5 mol% Ho^3+^-doped ZnO were found to be 2.032 and 2.067, respectively. Moreover, the 0.5 mol% Ho^3+^-doped ZnO sample exhibited an additional peak at about 3004 G (g = 2.24) and 2372 G (g = 2.83) whose features may originate from Ho^3+^. On the other hand, the peak-to-peak line widths (∆*H*) obtained were found to be 633, 648, 417 and 347 G for un-doped 0.25, 0.5 and 0.75 mol% Ho^3+^-doped samples, respectively. Furthermore, the number of spins (*N_s_*) contributing to the ferromagnetic resonance was found to be 1.576 × 10⁸, 2.906 × 10⁸, 3.35 × 10⁸ and 0.284 × 10⁸ for the 0, 0.25, 0.5 and 0.75 mol% Ho^3+^ samples, respectively (see Figure 8). It is worth mentioning that the observed enhanced ferromagnetism is related to the number of spins participating in the ferromagnetism and further related to the concentration of Ho^3+^ [64]. In fact, the maximum microwave absorption intensity was attained at the critical concentration of 0.5 mol%; above this concentration, a severe decrease was observed (see Figure 7). This observation could be attributed to the ferromagnetism saturation due to excess of holmium ions being segregated on the surface of the ZnO host matrix. ESR spectra of Ho^3+^-doped ZnO samples showed a similar intensity trend as compared to the UV–VIS transmittance spectra.

### 3.5. Time-Resolved Photoluminescence Lifetime Analysis

**Figure 9:**
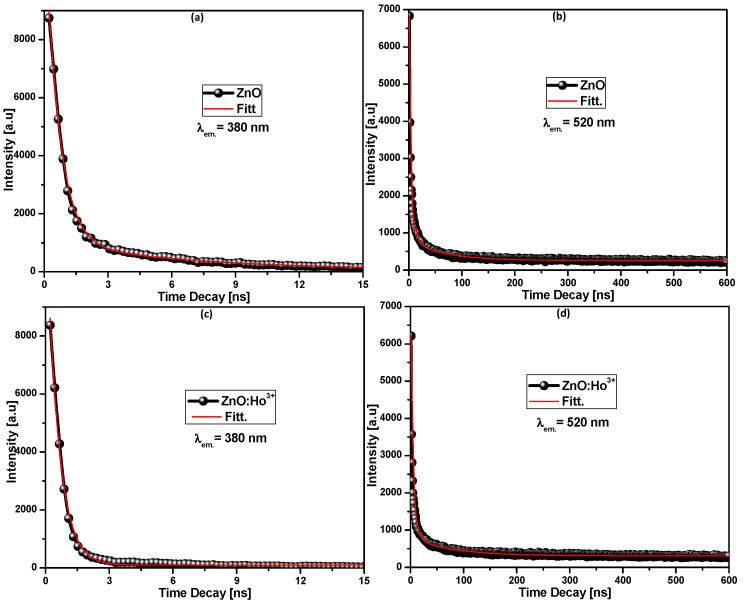
Exciton and defects TRPL lifetime decay of (**a**,**b**) ZnO and (**c**,**d**) ZnO:Ho^3+^ (0.5 mol%) nanostructures.

Time-resolved photoluminescence (TRPL) decay analysis of un-doped and 0.5 mol% Ho^3+^-doped ZnO samples, which focused on the exciton (380 nm) and defect (520 nm) emission is depicted in Figure 9a–d, and the related fitted parameters are presented in Table 2. The TRPL data collected were analyzedusing multiple exponential functions in DAS-6 software platform. The obtained TRPL decays were found to be non-exponential, meaning that several emissive states were present in all TRPL data with the exception of ZnO:Ho^3+^ (0.5 mol%) data which exhibited exponential behaviour at 380 nm (Figure 9c). The decay profile of ZnO exciton (380 nm) has been previously reported to contain two emissive states, a fast and slow component [76,77,78,79]. The fast decay component (*τ*_1_) has been assigned to non-radiative de-activation, and the slowdecaytrace (*τ*_2_) resulted from radiative lifetime of free-excitons [79]. However, in the case of ZnO defect emission (520 nm), the faster decay trace (*τ*_1_) has been assigned to arise from (i) radiative recombination of shallowly trapped electrons and deep trapped holes and (ii) the recombination of donor acceptor pair [77].

Moreover, due to the non-exponential nature of the TRPL decays under investigation, a bi- and tri-exponential function were the most reasonable approach to fit the data using the model presented equation below:

(5) $$I\left(t\right)=I_{0}\overset{n}{\underset{i=1}{\sum }}A_{i}e^{-\left(\frac{t}{\tau_{i}}\right)}$$

where I(t) is the fluorescence intensity at time *t*, $I_{0}$ is the initial fluorescence intensity, $\tau_{i}$ are lifetimes, and $A_{i}$ are pre-exponential factors.

By monitoring the emission at 380 nm (3.26 eV), it has been observed that the complete de-activation was achieved in about 15 ns, indicating the fast decay of the free-exciton transition. On the other hand, the 520 nm (2.38 eV) emission revealed the occurrence of a much longer decay due to the dominance of deep traps kind of defects. However, for both un-doped and 0.5 mol% Ho^3+^-doped samples, the best fit revealed the existence of three emissive states at 520 nm Table 2a,b. It is worth noting that the averaged slow decay observed in un-doped ZnO defect emission results from more efficient trapping of the charge carriers due to high density of defects [77].

Interestingly, the decay profile of the ZnO:Ho^3+^ (0.5 mol%) at 380 nm exhibited a single emissive state much faster than that of un-doped ZnO which contained two emissive states. This could be the result of the distortion in the band-structure induced by Ho^3+^ ions doping. Moreover, we can speculate on the behaviour of the slow decay observed in the TRPL at 380 nm, which completely vanishes, this phenomenon is indicative of an energy transfer from the ZnO exciton to Ho^3+^ [79]. This result is in accordance with the photoluminescence results reported earlier in this study. Finally, the longer decay of (*τ*_1_) observed in the doped sample indicates the good optical quality of ZnO:Ho^3+^ (0.5 mol%) as compared to the un-doped ZnO sample.

## 4. Conclusions

In summary, the use of the sol–gel method allows facile successful synthesis of Ho^3+^-doped ZnO (0.5 mol%) nanocrystals in which the Ho^3+^ ions were found to be emissive through the *4f-4f* electronic transitions. Holmium was actively emissive at about 540 (^5^S_2_,^5^F_4_→^5^I_8_), 574, 606, 670 (^5^F_5_→^4^I_8_) and 808 nm allied with the respective intra-ionic transitions. Moreover, optimal 0.5 mol% Ho^3+^ incorporation in ZnO relatively increased the crystallite size from 4 to 8 nm, tuned the band-gap and modified the initial morphology of un-doped ZnO spherical-like to rods-like which was attributed to Ostwald ripening effect. Furthermore, enhanced room temperature ferromagnestism related to the number of spins participating to the ferromagnetic resonance was reported. Finally, Time-resolved photoluminescence analysis revealed the change induced in the relaxation charge carriers in defects states as a result of 0.5 mol% Ho^3+^ doping in ZnO.

## Figures and Tables

**Table 1:** The XRD parameters and the average estimated crystallite size (D) from Scherrer equation.

Sample	2θ(101) [Degrees]	∆(2θ) [Rad] FWHM = β	D [nm]
Un-doped ZnO	36.52	0.02913	5.0
0.25 mol% Ho	36.34	0.02234	6.5
0.5 mol% Ho	36.55	0.01768	8.2
0.75 mol% Ho	36.55	0.02042	7.1

**Table 2:** (**a**) PL Decay of ZnO nanocrystals dispersed in ethanol under λ_ex._= 320 nm using NanoLED. (**b**) PL Decay of ZnO:Ho^3+^ (0.5 mol%) nanostructures dispersed in ethanol under λ_ex._ = 320 nm using NanoLED.

(**a**)				
λ_em._ (nm)	τ_1_ (ns)	τ_2_ (ns)	τ_3_ (ns)	χ^2^
380	0.651	6.769	-	0.99
520	0.828	7.085	65.257	0.99
(**b**)				
λ_em._ (nm)	τ_1_ (ns)	τ_2_ (ns)	τ_3_ (ns)	χ^2^
380	0.566	-	-	0.99
520	0.803	7.452	79.292	0.99

## Data Availability

Data is contained within the article or Supplementary Material.

## Supplementary Materials

The following are available online at https://www.mdpi.com/article/10.3390/nano11102611/s1, Figure S1: SEM image used to conduct EDS mapping, Figure S2: HRTEM images for (A) un-doped and (B) (0.5 mol%) Ho^3+^-doped ZnO nanocrystals.
