# Supplementary material for: Unveiling Semiconductor Nanostructured Based Holmium-Doped ZnO: Structural, Luminescent and Room Temperature Ferromagnetic Properties
Source: Nanomaterials (Basel). 2021 Oct 4;11(10):2611. doi: 10.3390/nano11102611 (PMC8537373; doi:10.3390/nano11102611)

Supporting Information (SI)

Unveiling Semiconductor Nanostructured Based Holmium-Doped ZnO Structural, Luminescent and Room Temperature Ferromagnetic Properties

G. L. Kabongo [1,*], G. H. Mhlongo [2] and M. S. Dhlamini [1]

[1] Department of Physics, University of South Africa, Pretoria P.O. Box 392, 0003, South Africa; dhlaminims@hotmail.com
[2] CSIR-National Centre for Nano-Structured Materials, Pretoria P.O. Box 395, 0001, South Africa; gmhlongo@csir.co.za
* Correspondence: geekale@gmail.com

Figure S1: SEM image used to conduct EDS mapping

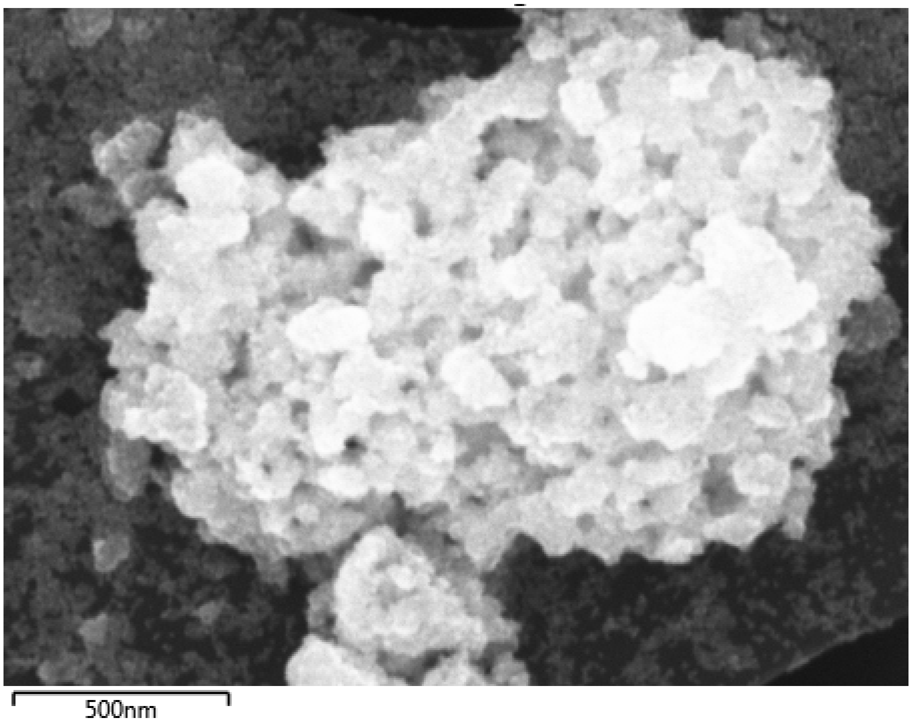

Figure S2: HRTEM images for (A) un-doped and (B) (0.5 mol%) Ho^{3+}-doped ZnO nanocrystals.

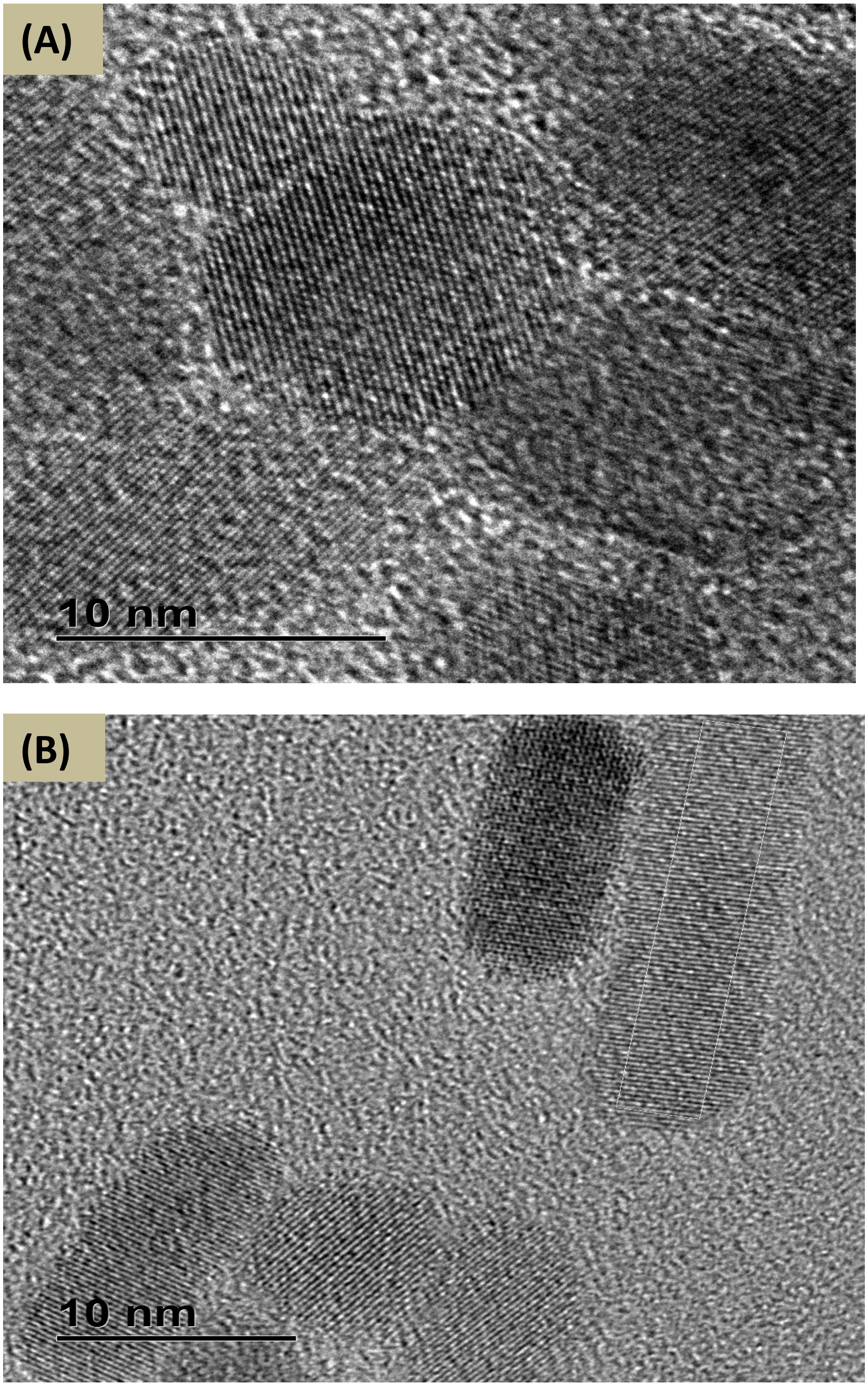

Supplement: Supplementary file 1 [file nanomaterials-11-02611-s001.zip › nanomaterials-1336877 Supplementary Materials - updated.pdf]
